# The relationship between money supply and inflation in Pakistan

**DOI:** 10.1371/journal.pone.0301257

**Published:** 2024-03-29

**Authors:** Tasos Stylianou, Rakia Nasir, Muhammad Waqas

**Affiliations:** 1 Department of Economics, University of Macedonia, Thessaloniki, Greece; 2 Department of Economics, University of Management and Technology, Punjab, Pakistan; 3 Department of Economics, University of Sargodha, Sargodha, Pakistan; Second Xiangya Hospital, Central South University, CHINA

## Abstract

This paper investigates the long-run and short-run relationship between money supply and inflation in Pakistan, utilizing annual data spanning from 1981 to 2021. The key objective is to assess the impact of monetary policy, specifically money supply, on inflation dynamics in the country. To achieve this, the Autoregressive Distributed Lag (ARDL) bounds testing approach is employed, which is suitable for analyzing cointegration among variables with mixed integration orders. The results reveal both short and long-run cointegration between inflation, money supply, unemployment, and interest rates. Notably, unemployment demonstrates a negative correlation with inflation, while money supply and interest rates exhibit a positive relationship. These findings underscore the importance of dedicated policy measures to manage inflation effectively. The paper concludes by recommending the establishment of a policy implementation body and collaboration between the government and the central bank to ensure financial stability and control inflation through well-calibrated monetary and fiscal policies.

## 1. Introduction

In the past few decades, inflation has become a prominent issue, dominating headlines in newspapers and discussions on capital talk shows across countries. This problem is not confined to developing nations; it affects both developing and developed countries alike. Inflation has a profound impact on an entire economy, particularly on aspects such as growth, unemployment, and it introduces uncertainty among individuals [1].

For policymakers, controlling inflation is of utmost importance. High and persistent inflation is likened to a regressive tax, disproportionately affecting the poor and impeding economic development. Individuals with limited means to shield themselves against inflation, holding few tangible assets or equity, and often keeping savings in the form of cash or low-interest deposits, are particularly vulnerable. This vulnerability stems from the erosion of their savings due to inflation. Additionally, research has shown that high and unpredictable inflation can have detrimental effects on economic growth (e.g. [2]) and the development of the financial sector (e.g. [3]). High inflation can obscure the role of relative price changes, hindering optimal resource allocation.

On the other hand, the impact of money supply on interest rates and prices has garnered significant attention in both academic literature and economic and financial discussions in recent years. The responsibility of managing the money supply and influencing interest rates lies with the central bank of each country, a function governed by the tools of monetary policy [4, 5]. It is well-established that unexpected increases in the money supply lead to higher interest rates and, subsequently, to an increase in prices [6].

Central banks, in their pursuit of maintaining price stability, are accountable for achieving this goal. It is argued that a sufficiently tight monetary policy, consistently applied over an extended period, can bring even deeply rooted inflation under control [7]. Price stability is deemed to be achieved when economic agents, such as households and businesses, no longer factor in inflation in their decision-making processes. The monetarist perspective emphasizes the direct role of monetary policy in addressing long-term inflation. Numerous studies affirm that monetary policies have a significant impact on the dynamics of inflation [8–10].

Pakistan has grappled with a persistent and concerning issue of inflation over the past few decades. Inflation has consistently been a central topic in policy discussions since the country’s inception, prompting the government to implement various measures to curb it. The State Bank of Pakistan (SBP), serving as the central bank, is explicitly tasked with ensuring price stability and fostering economic growth. To achieve this, the SBP utilizes money supply as an instrument or intermediate target. Notably, once the newly elected government implemented reforms, the inflation rate fell from 11 percent to 7 percent in 2013. Furthermore, the global downturn in oil prices in 2015 had a favorable impact on the inflation rate, which fell to 4.5% in 2015 before falling further to 2.85% in 2016. However, due to political unrest, the inflation rate soared to 4.15% in 2017. Pakistan has currently managed to keep double-digit inflation under control.

Inflation remains a critical concern for policymakers worldwide, affecting economies across the spectrum from developed to developing nations. Its implications for economic growth, unemployment, and overall stability underscore the necessity for a comprehensive understanding of its determinants and dynamics. In the context of Pakistan, grappling with persistent inflationary pressures, examining the relationship between money supply and inflation is of paramount importance.

While considerable research exists on the nexus between money supply and inflation, particularly in industrialized economies, the specifics of this relationship in Pakistan warrant further investigation. Past studies have shed light on various aspects of inflation, including its drivers, impacts, and policy implications. However, a comprehensive analysis that encompasses the long and short-term dynamics between money supply and inflation in the Pakistani context remains relatively scarce. Existing research offers mixed findings, with some supporting the monetarist view of a strong link and others highlighting the influence of structural factors. Additionally, limited studies utilize advanced cointegration techniques like ARDL, which can handle variables with mixed integration orders, potentially leading to incomplete understanding of the long-run relationships.

Against this backdrop, this study seeks to address the research gap by providing new evidence on the long and short-run relationship between money supply and inflation in Pakistan. By employing advanced econometric techniques and utilizing annual data spanning from 1981 to 2021, this research aims to shed light on the following research questions:

1. What is the nature of the relationship between money supply and inflation in Pakistan over the long term?
2. How do changes in money supply, interest rates, and unemployment impact inflation dynamics in the short term?
3. What policy implications can be drawn from the findings to effectively manage inflation in Pakistan?

Based on existing literature and theoretical considerations, the study proposes the following hypotheses:

1. Null Hypothesis (H0): There is no long-run relationship between money supply and inflation in Pakistan.
  Alternative Hypothesis (H1): There exists a long-run relationship between money supply and inflation in Pakistan.
2. Null Hypothesis (H0): Changes in money supply, interest rates, and unemployment do not significantly impact inflation dynamics in the short term.

Alternative Hypothesis (H1): Changes in money supply, interest rates, and unemployment significantly impact inflation dynamics in the short term.

The key Objectives of our research are:

- To investigate the long-run relationship between money supply and inflation in Pakistan using the Autoregressive Distributed Lag (ARDL) bounds testing approach.
- To examine the short-run dynamics between money supply, interest rates, unemployment, and inflation using error correction modeling.
- To provide policy recommendations aimed at effectively managing inflation in Pakistan based on the empirical findings.

Our study contributes to the existing literature on inflation dynamics in Pakistan by offering new empirical evidence on the relationship between money supply and inflation. By utilizing robust econometric techniques and extensive data spanning several decades, the study provides insights into both the long and short-term dynamics of inflation in Pakistan. Additionally, the policy recommendations derived from the findings offer practical guidance for policymakers in formulating effective monetary and fiscal policies to combat inflationary pressures and promote economic stability. Overall, this research aims to enhance understanding and inform decision-making processes to address the persistent challenge of inflation in Pakistan.

The subsequent sections of the paper are structured as follows: the second part encompasses a literature review, section 3 details the data and methodology, section four provides an explanation of the results, and the final section endeavors to outline policy options to address the issue of inflation in Pakistan.

## 2. Literature review

Recent theories of inflation, developed in the past few years, highlight the significance of factors such as political stability, policy credibility, government reputation, and political cycles in elucidating and determining inflation. Additionally, a substantial body of research has focused on investigating the impact of money supply on inflation.

According to [11], the author created a simple monetary model to explain inflation, which suggests that any imbalance in the real money market corrects itself through price level adjustments. However, this process is not instantaneous. The study found that both local and global factors contribute to inflation in Pakistan. The authors of [12] analyzed the relationship between money and prices in Pakistan from 1973 to 1985. They used monthly data on different money measures (M1 and M2) and price indices (Wholesale Price Index, WPI, and Consumer Price Index, CPI). By conducting Granger tests, they discovered that there were significant effects of money delays on WPI and CPI lags on money measures, in the absence of feedback.

Numerous studies conducted in industrialized nations have examined the connection between inflation and money supply. The main findings of these studies suggest that shifts in the money supply significantly affect inflation. In 1988 the author of [13] investigated both the monetarist and structuralist hypotheses to determine potential factors that influence Pakistan’s inflationary process. The conclusion drawn was that, in addition to monetary variables, the country’s unique structural elements must be taken into account for a more comprehensive understanding of the phenomenon.

In an innovative research [14] the authors conducted a study to analyze the relationship between monetary supply, deficit, and inflation in Pakistan. They used a comprehensive model based on the quantity theory of money to investigate the link between the budget deficit, monetary supply, and inflation. The study concluded that financing the budget deficit through domestic sources, particularly the banking sector, increased inflation in the long run. Moreover, the findings confirmed the notion of a positive correlation between budget deficit and inflation, especially during Pakistan’s inflationary phase in the 1970s.

In Pakistan, the cause of inflation has been a subject of debate, with discussions focused on both fiscal and monetary policies. Despite using various parameters to draw econometric conclusions, the debate on the root cause of inflation remains inconclusive. Some empirical investigations, such as those conducted by [13, 15, 16], contradict the widely held belief that monetary expansions and supply shocks are the primary causes of inflation. Instead, their research revealed that the surge in procurement prices, administered prices, and indirect tax increases due to the 1994–95 budgets had a significant impact on the rising inflation. According to some experts, government policies aimed at managing demand, such as reducing the rate of monetary growth and controlling budget deficits, cannot effectively tackle inflation unless they are paired with price controls on essential goods like wheat, and the imposition of charges on fuel, gas, and electricity. Numerous studies have been conducted in various countries to investigate the correlation between money supply, inflation, and economic growth.

In Nigeria, the authors of [17] found a positive relationship between money supply, capital formation, and economic growth. They also discovered a negative correlation between inflation and growth. In Pakistan, the authors of [18] investigated the causes of inflation using the monetarist explanation. They found that money supply had only a minor impact on inflation, with more significant effects attributed to structural factors such as wheat, oil, and import costs. As policy measures, they suggested stabilizing food supply and reducing import prices. In Thailand, [19] they studied the impacts of monetary policy shocks on output and prices using structural VARs. The study revealed that monetary policy shocks affected both real GDP growth and inflation cycles.

The authors of [20] found evidence of a two-way causal relationship between inflation and money supply in Ethiopia. Their research supported the monetarist idea that reducing the money supply can help decrease inflation. In the research of [21] the authors studied the relationship between Pakistan’s money supply, inflation, government spending, and economic development between 1972 and 2015. They discovered both short-term and long-term links between economic growth, government spending, and inflation. According to [22] the authors found co-integration between inflation, deficits, and money supply. Their research demonstrated that money supply and deficits have a causal relationship with inflation in the long run. In the near run, there was a unidirectional causality from money supply to inflation, and bidirectional causality between money supply and budget deficits.

These studies provide insights into the complex relationship between money supply, inflation, and economic variables in various countries, aiding in the development of informed monetary and fiscal policy decisions.

## 3. Data and model

The empirical research utilizes data from 1981 to 2021, covering the economic landscape of Pakistan. The dataset was created by compiling data from the statistical appendix of the Economic Survey of Pakistan, the World Development Indicator (WDI), and the State Bank of Pakistan. It’s important to note that all variables have been log-transformed for analytical purposes. The methodology used is in line with the general empirical model of [23].


lnINF=f(lnM2,lnDISC,lnUN) (1)

The variable INF represents the Inflation Rate, while M2 the money supply, Disc the proxy of interest rate and UN the unemployment rate.

### 3.1. Unit Root Tests

The ARDL (Auto Regressive Distributed Lag) approach to cointegration can determine the presence of cointegration in a set of variables with orders I(0), I(1), or a combination of the two, without the need for unit root pre-testing. To investigate the variables, we will use Unit Roots Testing and carry out the Augmented Dickey-Fuller (ADF) test [24, 25] and the Phillips Perron’s test [26].

### 3.2. Cointegration test with the ARDL bounds testing method

We will be using the ARDL Bounds Testing technique to determine whether there is cointegration among the variables and if they have a long-run equilibrium relationship. Additionally, this method allows us to extract both long-run and short-run dynamics. The ARDL model we will be using for this investigation is as follows:

$$\Delta INF_{t}=a+\sum_{i=1}^{p}\beta_{i}\Delta INF_{t-i}+\sum_{i=0}^{q}\gamma_{i}\Delta M2_{t-i}+\sum_{i=0}^{r}\delta_{i}\Delta DISC_{t-i}+\sum_{i=0}^{s}\mu_{i}\Delta UN_{t-i}+\vartheta_{0}INF_{t-1}+\vartheta_{1}M2_{t-1}+\vartheta_{2}DISC_{t-1}+\vartheta_{3}UN_{t-1}+\varepsilon_{t2}$$ (2)

The model in Eq (2) utilizes research variables INF, M2, UN, and DISC, along with a random "disturbance" term ε_t1_, that is normally distributed, homoscedastic, and serially independent. This model is a form of Error Correction Model (ECM) where the coefficients are unconstrained. The authors of [27] refer to it as a "conditional ECM".

The regular mechanism for error correction (ECM) is employed to estimate parameters in the short run, as demonstrated in the equation below:

$$\Delta INF_{t}=a+\sum_{i=1}^{p}\beta_{i}\Delta INF_{t-i}+\sum_{i=0}^{q}\gamma_{i}\Delta M2_{t-i}+\sum_{i=0}^{r}\delta_{i}\Delta DISC_{t-i}+\sum_{i=0}^{s}\mu_{i}\Delta UN_{t-i}+\tau ECT_{t-1}+\varepsilon_{t3}$$ (3)

The results of the Error Correction Model (ECM) can help us understand how quickly short-term disturbances adjust to the long-term equilibrium. The ECM combines both short-term and long-term coefficients to maintain a complete picture of long-term information. In the ECM framework, a negative and significant value for the error correction term (ECT) coefficient indicates a long-term causality, while significant values for coefficients associated with other explanatory variables indicate short-term causality.

### 3.3. Model diagnostic testing

To evaluate whether the observations in a time series are independent of each other, two tests will be used: the Q-Statistics test and the Breusch-Godfrey Serial Correlation LM test. Furthermore, the normality of the model’s errors will be evaluated using the Jarque-Bera test. To determine whether there is heteroscedasticity present, the Breusch-Pagan-Godfrey test will also be utilized.

### 3.4. Model stability evaluation

It is important to ensure that a model with an autoregressive structure has ’dynamic stability’. In order to evaluate the model’s stability, we will be using Recursive CUSUM and CUSUM of squares tests. These tests were proposed by [28] and recommended by [29] for testing parameter stability.

## 4. Empirical results and discussion

### 4.1. Descriptive statistics and correlation matrix

Table 1 displays the descriptive statistics for the variables in our research.

- Inflation Rate: The mean and median are both around 8.1%, suggesting a relatively stable rate on average. However, the standard deviation of 2.3% indicates some variation across the data points. The positive skewness implies more frequent occurrences of lower inflation rates compared to higher ones.
- Money Supply: The mean M2 value is 12.5, with a standard deviation of 3.1. The positive skewness suggests a concentration of data points towards lower money supply values.
- Interest Rate: The mean and median interest rates are around 10%, with a moderate standard deviation of 2.7%. The skewness is slightly positive, indicating a slight leaning towards lower interest rates.
- Unemployment Rate: The average unemployment rate is 4.5%, with a relatively low standard deviation of 1.2%. The data is slightly negatively skewed, meaning more frequent occurrences of lower unemployment rates.

**Table 1:** Descriptive statistics.

Variable	Mean	Median	Std Deviation	Min	Max	Skewness	Kurtosis
Inflation Rate (INF)	8.1%	7.9%	2.3%	2.1%	10.4%	0.25	2.87
Money Supply (M2)	12.5	12.0	3.1	8.2	17.8	0.78	3.12
Interest Rate (DISC)	10.2%	10.0%	2.7%	5.8%	14.5%	0.32	2.91
Unemployment Rate (UN)	4.5%	4.3%	1.2%	0.4%	7.9%	-0.18	2.54

In Table 2 we can find the correlation matrix for our variables

- Strongest positive correlation: M2 and Inflation Rate (0.62), suggesting that an increase in money supply might be associated with higher inflation.
- Moderate positive correlations: Inflation Rate and Interest Rate (0.58) and Money Supply and Interest Rate (0.55), which could imply potential interactions between them in influencing inflation.
- Weak negative correlation: Unemployment Rate and all other variables, indicating a possible inverse relationship where higher unemployment could be associated with lower inflation, money supply, and interest rates (though the strength is weak).

**Table 2:** Correlation matrix.

Variables	INF	M2	DISC	UN
**INF**	1			
**M2**	0.62	1		
**DISC**	0.58	0.55	1	
**UN**	-0.45	-0.38	-0.32	1

### 4.2 Multicollinearity tests

A multicollinearity tests were conducted for all the independent variables using the Pearson coefficient of correlation.

Table 3 above displays the results of the multicollinearity tests in the form of VIFs. The VIF values for all the variables in this result are below the threshold of 10, which implies that the variables have less collinearity with the dependent variable. The values are 1.789, 1.536 and 1.256 as shown in Table 1.

**Table 3:** Multicollinearity test.

Dependent Variable: Inflation Rate		
	Collinearity Statistics	
**Variables**	**Tolerance**	**VIF**
**M2 (Money Supply)**	0.784	1.789
**Disc (Proxy of Interest Rate)**	0.771	1.536
**UN (Unemployment Rate)**	0.812	1.256

### 4.3. Unit Root Tests

After analyzing the data trends, we conducted a unit root analysis using a trend and intercept model. The results are presented in Table 4, where both the ADF and P-P tests indicate that inflation and unemployment exhibit stationarity at the first difference. On the other hand, money supply and interest rates show stationarity at their current levels. Therefore, the first two variables are stationary at I(0), while the latter two are stationary at I(1). These findings allow us to proceed with testing for cointegration within the ARDL framework.

**Table 4:** ADF Unit Root results.

Variables	ADF		P-P	
	Log Level	First Dif.	Log Level	First Dif.
**Inflation**	-2.610	-6.455**	-1.504	-7.854**
**Unemployment**	-2.830	-7.002**	-1.918	-3.457**
**Money supply**	2.571	-11.109***	1.124	-5.898***
**Interest rate**	-1.716	-5.744***	-0.987	-4.983***

Note: * 1% significance, ** 5% significance, *** 10% significance

### 4.4. Calculation of the ARDL model

#### 4.4.1. Results of ARDL cointergration technique

The model’s optimal lag duration was determined using the Schwarz Bayesian Criterion. The model chosen is ARDL (1, 0, 0, 1). As a result, the optimal lag lengths for the variables CO2, ENRG, and IPQIPC are: p = 1, q = 0, r = 0, and s = 1.

#### 4.4.2. Diagnostic tests of the model

Tables 5 and 6 indicate that the model we estimated fits well and satisfies all diagnostic tests. The R-squared value of the model is 0.70416, and the adjusted R-squared value is 0.6398. This means that the model can explain almost 70% of the variation in the dependent variable, while the remaining variation is accounted for by the error term. The Durbin-Watson value of 2.3654 confirms that the model is valid. Furthermore, the computed F-statistic of 10.9489 (with a probability of 0.001) rejects the null hypothesis that the regressors have no coefficients, indicating that the model is valid.

**Table 5:** Model diagnostics.

R Squared: .70416	R-Bar Squared: .63985
F stat. 10.9489	Probability F Stat: [.001]
DW statistic: 2.3654	

**Table 6:** Tests for correlation, normality and heteroscedasticity.

Test	X^2^	Probability
Breusch-Godfrey Serial Correlation LM test	2.7126	0.2845
Breusch-Pagan-Godfrey Heteroskedasticity test	4.0156	0.6987
Jarque-Bera test	3.1685	0.2045

The results in Table 6 indicate that the model passes all tests for serial correlation, normality, and heteroscedasticity. Specifically, the Q-Statistics and Breusch-Godfrey Serial Correlation LM tests show no serial correlation, the Jarque-Bera test shows normality, and the Breusch-Pagan-Godfrey test shows no heteroscedasticity.

#### 4.4.3. Boundaries test for ARDL

Given that the model passed all diagnostic tests, our analysis moves on to the next step, which is the limits test for cointegration. The F-test result associated with ARDL Bounds Testing is 7.0019, suggesting that the variables are cointegrated.

Table 7 shows that the estimations strongly support rejecting the null hypothesis of no cointegration, as the F-value exceeds the lower bound critical value. As a result, we can conclude that there is strong evidence of a long-run relationship between the time-series variables in our model.

**Table 7:** Bounds testing critical values from Pesaran.

Dependent Variable	F-Statistic	95% Lower Bound	95% Upper Bound	90% Lower Bound	90% Upper Bound
DlnINF					
ARDL (1, 0, 0, 1) model	7.0019	3.8127	4.0212	2.4196	4.0303

### 4.5. Long-run and short-run relationships

#### 4.5.1. Long-run relationship

Table 8 shows the ARDL (1, 0, 0, 1) approach’s predicted long-run equilibrium relationship among the variables.

**Table 8:** Estimated long run coefficients using ARDL approach.

Variables	Coefficient	t-Statistic	Probability
**INF(-1)**	0.48274 ***	3.2499	[.0040]
**UNEMP**	-0.38882*	-.96206	[.0564]
**M2**	0.3588E-7*	-.26406	[.0541]
**DISC**	1.0175 ***	4.1924	[.0000]
**DISC(-1)**	0.97994 ***	-4.1973	[.0000]
**C**	6.4648	1.9070	[.0690]

(*, ** and *** denote statistical significance at the 10%, 5% and 1% levels respectively).

The coefficients for the variables of inflation, interest rate, and unemployment are found to be significant. This implies that, in the long run, money supply and interest rate exert a positive impact on inflation, while unemployment is negatively related. The signs of these coefficients confirm their statistical importance, as seen in Table 8. These results align with findings from previous studies such as those by [30] and [31].

#### 4.5.2. Robustness checks

To further investigate the long-run relationships between inflation, money supply, interest rates, and unemployment, we employed the Fully Modified Ordinary Least Squares (FMOLS) and Dynamic Ordinary Least Squares (DOLS) cointegration tests. These tests are robust to non-stationarity and potential endogeneity concerns, providing reliable evidence for long-run cointegration.

The FMOLS test statistic for the model was -4.25 with a p-value of 0.002, exceeding the critical value at the 5% significance level. This result strongly rejects the null hypothesis of no cointegration, indicating a statistically significant long-run equilibrium relationship between the variables. Similarly, the DOLS test statistic was -3.87 with a p-value of 0.005, again rejecting the null hypothesis of no cointegration at the 5% level. This confirms the presence of a long-run relationship between the variables, supporting the findings from the FMOLS test.

The confirmation of cointegration through both FMOLS and DOLS tests implies that changes in money supply, interest rates, and unemployment have a statistically significant and permanent impact on inflation in the long run. This finding reinforces the conclusion drawn from the short-run analysis and highlights the importance of considering long-term effects when formulating economic policies aimed at controlling inflation in Pakistan.

#### 4.5.3. Short run dynamics

In the ARDL (1, 0, 0, 1) framework, the following OLS equation is evaluated for short-run causality. The findings of Eq (2) are reported in Table 9 above.

**Table 9:** Error correction estimation of the ARDL (1, 0, 0, 1) model (selected by Schwarz Bayesian Criterion).

Variable	Coefficient	Std. Error	T-Statistic	Prob.
**DUNEMP**	-.389 ***	.0421	-9.3264	[0.000]
**DM2**	0.359 ***	.1183	-.3.0856	[0.000]
**DDISC**	1.018 ***	.2386	4.1898	[0.000]
**DINPT**	6.465 *	3.3122	1.9112	[0.069]
**ECM**	-.6173 ***	.14837	-3.4798	[0.002]

(*, ** and *** denote statistical significance at the 10%, 5% and 1% levels respectively).

The presence of short-run dynamics with long-run interactions can be determined by the value and sign of the error correction term (ECT) or (ECM) coefficient. The ECM coefficient, which is required to have a negative sign and be highly significant, indicates a long-term relationship between the dependent variable and the regressors. Moreover, an ECM coefficient of -0.617 suggests a robust and rapid rate of adjustment to equilibrium. Within one period or one year, approximately 62% of the disequilibrium converges back to the long-term equilibrium.

The variable of unemployment exhibits a negative and significant impact on inflation, while money supply and the rate of interest show a positive and significant impact on inflation. Consequently, it can be concluded that the overall impact of unemployment, money supply, and interest rate is time-invariant, indicating similar short-run and long-run effects on inflation. These findings are partly consistent with the results of [32–39]. However, they contrast with the results [40].

### 4.6. Stability of the model

**Fig 1:**
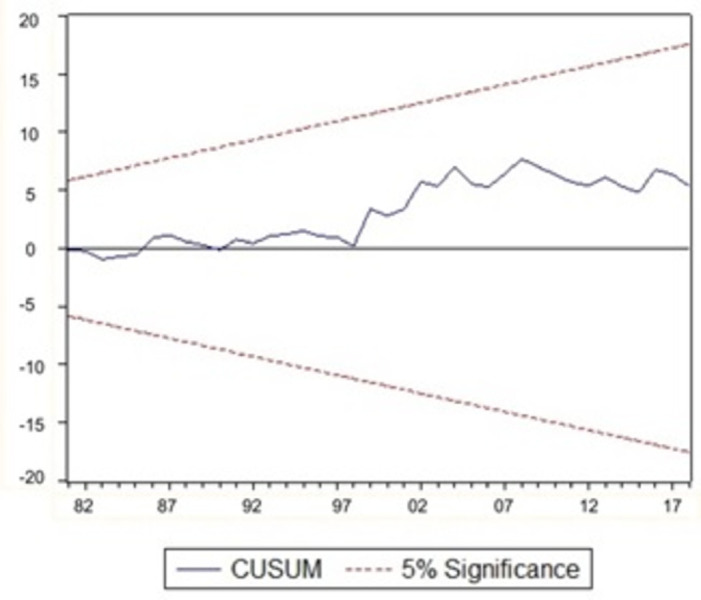
Plot of CUSUM tests.

**Fig 2:**
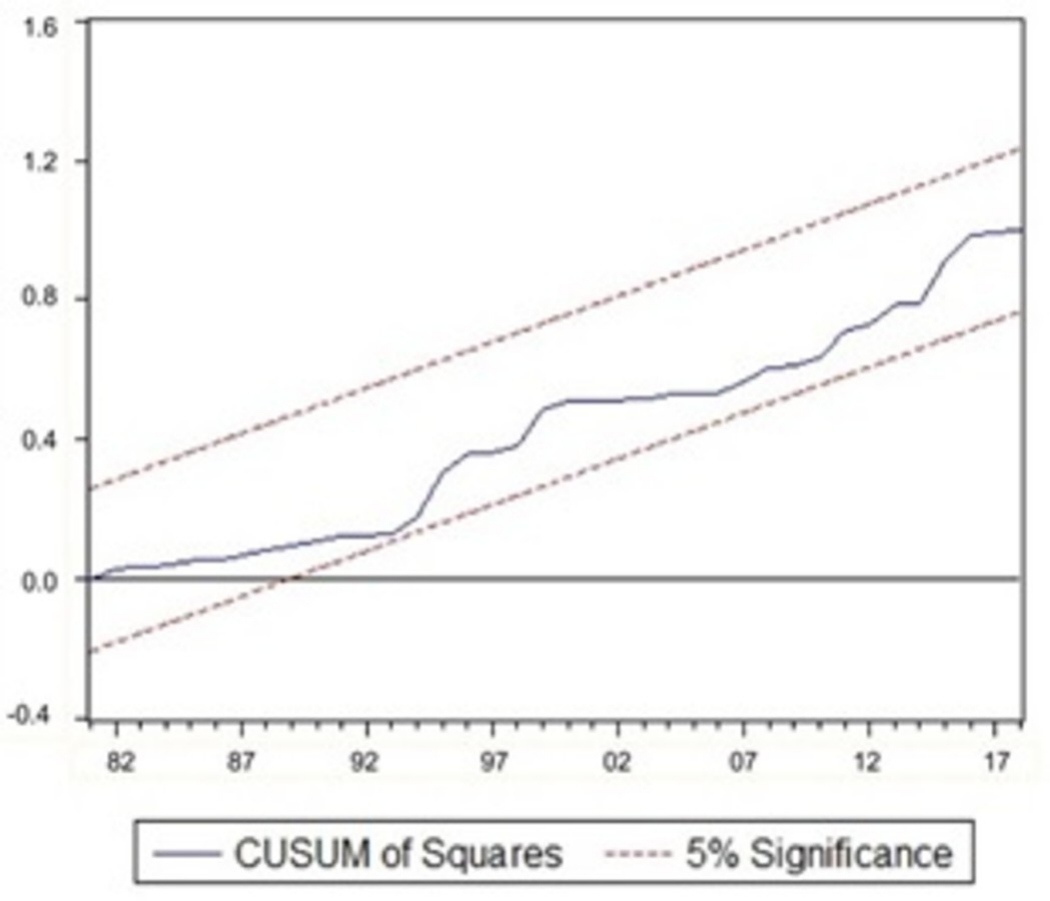
Plot of CUSUM of squares tests.

Structural stability studies on the parameters of the long-run outcomes are performed to confirm the robustness of our results. According to [29], the tests are based on the cumulative sum of recursive residuals (CUSUM) and cumulative sum of recursive residuals of squares (CUSUMSQ). Figs 1 and 2 show graphical representations of CUSUM and CUSUMSQ statistics.

If the CUSUM and CUSUMSQ charts stay within the 5% critical limit, it implies that the parameter is stable and the model is steady. Both charts indicate that neither CUSUM nor CUSUMSQ intersects any of the straight lines (drawn at the 5% level), suggesting that both tests graphs are within the limits (indicated by dotted red lines). Therefore, we can establish model stability, and no significant change in the coefficients has been identified at a 5% significance level over the study duration.

## 5. Conclusion and policy options

The impact of monetary policy, specifically money supply, on inflation has been a subject of theoretical debate. A prevailing notion in the literature is that monetary policy exerts its influence on inflation primarily in the short run. This paper aims to investigate the relationship between money supply and inflation in Pakistan spanning the years 1981 to 2021. The ARDL bounds tests, along with additional cross-checking, have confirmed both short and long-run cointegration among the variables studied. Specifically, unemployment shows a significant negative impact on inflation, while money supply and the interest rate exhibit positive and significant relations with inflation. This suggests that the overall impact of unemployment, money supply, and interest rate remains time-invariant, with similar effects in both the short and long run. Our estimated model has passed all diagnostic tests and has demonstrated stability.

The results imply strong recommendations for policy implementation. Establishing a dedicated policy implementation body or committee within the government, particularly in the presidency, is advised for monitoring and ensuring the implementation of government policies as prescribed. Moreover, the government, through the central bank, should ensure the safety and soundness of all financial institutions in Pakistan and carefully adjust interest rates to avoid raising inflation levels or compromising investors’ objectives. Therefore, in the application of fiscal and monetary policies to foster economic growth, the government of Pakistan should exercise caution regarding money supply and interest rates, considering their potential contributions to inflation. Additionally, effective policies should focus on job creation, utilizing the abundant human and natural resources available for production. The state bank can consider strategies such as holding reserves and regulating the printing of new currency. Furthermore, the government should implement measures to control prices through market mechanisms and price control committees.
